# Association Between Smoking and Amnesia in Southwest Iran: A Population-Based Cross-sectional Study

**DOI:** 10.34172/jrhs.2025.182

**Published:** 2025-04-01

**Authors:** Bahman Cheraghian, Zahra Rahimi, Seyed Jalal Hashemi, Amin Torabipour

**Affiliations:** ^1^Department of Biostatistics and Epidemiology, School of Public Health, Ahvaz Jundishapur University of Medical Sciences, Ahvaz, Iran; ^2^Alimentary Tract Research Center, Clinical Sciences Research Institute, School of Medicine, Ahvaz Jundishapur University of Medical Sciences, Ahvaz, Iran; ^3^Social Determinants of Health Research Center, Ahvaz Jundishapur University of Medical Sciences, Ahvaz, Iran

**Keywords:** Smoking, Amnesia, Adult, Iran

## Abstract

**Background:** Amnesia is a cognitive impairment that manifests as a deficit in the retrieval of previous memories and the acquisition of novel information. Limited research, especially in Iran, exists on the risk factors of amnesia, and smoking might be linked to a greater likelihood of experiencing memory issues and cognitive decline, including amnesia. The aim of this study was to explore the risk factors associated with amnesia and the connection between smoking and amnesia.

**Study Design:** A population-based cross-sectional study.

**Methods:** This study was conducted at the baseline of the Hoveyzeh cohort study on adults aged 35–70 years in southwest Iran between 2016 and 2018. The required data on socioeconomic factors, demographic characteristics, history of stroke, history of epilepsy, and history of head trauma were collected from the participants. The relationship between smoking and amnesia was assessed, and multiple logistic regression was employed to account for potential confounding variables.

**Results:** The mean age of the participants was 48.83±9.20 years, and 39% were male. The overall prevalence of amnesia was 4.2% (95% confidence interval [CI]: 3.8–4.6). The odds of having amnesia were significantly higher in smokers than in nonsmokers (adjusted odds ratio=1.52 [95% CI: 1.21–1.91]). Additionally, several other factors, including age, education level, type of residence, history of stroke, epilepsy, and history of trauma, were associated with amnesia.

**Conclusion:** Our investigations revealed a direct correlation between smoking and amnesia. To gain a more comprehensive understanding of the underlying mechanisms of these associations, it will be imperative to conduct future longitudinal studies.

## Background

 Amnesia is a memory disorder characterized by the inability to recall past experiences and acquire new knowledge, suggesting a disturbance of hippocampal function.^1^ It originates in the limbic system, a brain section that maintains memory.^2^ Transient global amnesia (TAG) is the most common cause of acute amnesia and is characterized by profound anterograde and retrograde amnesia, typically lasting up to 24 hours.^1^

 TAG is a multifactorial disease with several risk factors, including smoking^3^ traumatic head injuries,^4^ alcohol use,^5^ age, epilepsy, history of migraine,^6^ and cardiovascular diseases, such as high blood pressure and stroke.^7^

 According to the World Health Organization’s global report, by 2025, the smoking prevalence will be 8% in women and 30% in men.^8^ Despite early studies indicating that smoking may have beneficial effects on cognition, more recent evidence clearly shows that active smoking has neurotoxic effects on the brain and may indirectly increase the risk of cognitive impairment.^9^ Smoking is a significant risk to the health of the central nervous system, especially the brain. Evidence represents that nicotine is the main mind-altering ingredient in tobacco and is responsible for its addictive effects and worsening of memory function, especially at sustained concentrations.^10,11^ Some studies have reported that smoking negatively affects cognitive abilities, task performance, flexibility, memory, and sleep quality.^12^ Additionally, research has shown that adults with a history of nicotine abuse suffer more from cognitive impairments and visuospatial working memory than smokers who have quit smoking.^13,14^

 The trigger and risk factors for memory disorders are mostly unclear.^15^ To the best of our knowledge, few studies have assessed the impact of cigarettes on memory disorders in the Iranian population.^16^ Therefore, the present study was designed to identify possible risk factors for amnesia and evaluate the association between amnesia and smoking in a population-based cross-sectional study.

## Methods

###  Study design and participants

 This population-based cross-sectional study included 10,009 adults aged between 35 and 70 years who participated in the enrollment phase of the Hoveyzeh Cohort Study (HCS).^17^ The HCS is a site of the Prospective Epidemiological Research Studies in Iran (the PERSIAN Cohort Study).^18^ The study protocol was approved by the Ethics Committee of Ahvaz Jundishapur University of Medical Sciences. The inclusion criteria consisted of being aged 35-70 years, not having severe mental disorders, and being able to respond to questionnaires independently. However, participants with missing information on amnesia were excluded from the study. Finally, 9736 people underwent analysis.

###  Covariates

 In the present study, two indicators, including the education level as an individual-level indicator and the wealth index as a household-level indicator, were applied to evaluate socioeconomic status (SES). Several steps can be taken to calculate the wealth index based on household assets, including (1) identifying the variables to include, (2) assigning weights to each variable, (3) standardizing the variables, and (4) estimating the wealth index. First, a wealth index is identified, which typically includes household assets and characteristics correlated with wealth (e.g., ownership of a house, car, or other valuable assets), access to essential services (e.g., electricity or water), and the number of rooms per capita. The next step is to assign weights based on their relative importance in determining household wealth. This process can be performed through statistical techniques (e.g., principal component analysis or factor analysis) that identify underlying patterns in the data and assign weights accordingly. Moreover, to ensure that each variable is on the same scale, it is important to standardize them. This step can be taken by subtracting the mean and dividing it by the standard deviation of each variable. Finally, the wealth index can be computed by adding the weighted and standardized variables for each household. This process results in a composite score representing the household’s wealth. The wealth score was categorized into five groups, from poorest to richest, based on the quintiles.^19,20^

 The other covariates were age groups (35-39, 40-44, 45-49, 50-54, 55-59, 60-64, or ≥ 65 years), gender (male or female), marital status (single, married, widow, or divorced), residence type (urban or rural), educational level (illiteracy, primary school, secondary school, high school diploma, or university), and history of stroke (yes/no), epilepsy (yes/no), and head trauma (yes/no).

###  Cigarette consumption

 In our study, a smoker was defined as an individual who has smoked at least 100 cigarettes in their lifetime.^17^ Additionally, cigarette consumption in terms of “pack-years” was calculated to assess the dose-response relationship between smoking and amnesia. A pack-year is used to quantify the number of cigarettes smoked over a lifetime, with a pack equivalent to 20 cigarettes. It is measured by multiplying the number of packs smoked per day by the number of years the individual has smoked.^21^

###  Assessment of amnesia

 All the participants were asked to assess amnesia with a question: *“Have you ever experienced a memory disorder lasting more than a week?” *The response was either “yes” or “no” based on self-reporting.

###  Statistical analysis

 Means and standard deviations (SD), as well as frequencies and percentages, were used to report quantitative and qualitative variables, respectively. The chi-square test and chi-square test for trend were employed to analyze the relationship between categorical variables. To assess the association between amnesia and smoking while controlling for confounding variables, the logistic regression model was utilized to calculate both the crude and adjusted odds ratios (ORs). A *P* value of < 0.05 was considered statistically significant. Eventually, SPSS (version 22.0) was employed for data analysis.

## Results

 A total of 9736 participants were included in this study. The mean ± SD of the participants’ age was 48.83 ± 9.20 years, and 39% (n = 3795) were male.

 The results of the chi-square test are provided in Table 1. A direct association was observed between amnesia and age (*P*< 0.001), while education was inversely related to amnesia (*P*< 0.001). In urban areas, the disease was more prevalent than in rural areas (*P*= 0.049). Additionally, amnesia was significantly higher in smokers (*P*< 0.001). Amnesia significantly increased as the pack years of cigarette consumption increased (*P* for trend < 0.001), and participants had a history of stroke (*P*< 0.001), trauma to the head (*P*< 0.001), or epilepsy (*P*= 0.009). Based on the results, no significant association was found between gender, marital status, or wealth status and amnesia (*P* > 0.05).

**Table 1:** Association between amnesia and demographic characteristics, socioeconomic status, and several disease risk factors

**Variables**	**With amnesia**		**Without amnesia**		***P*** ** value**
	**Number**	**Percent**	**Number**	**Percent**	
Age group (y)					
35-39	44	2.4	1786	97.6	0.001
40-44	66	3.4	1895	96.6	
45-49	83	4.7	1675	95.2	
50-54	67	4.6	1386	95.4	
55-59	58	4.6	1195	95.4	
60-64	46	5.9	781	94.1	
≥ 65	44	6.3	656	93.7	
Gender					
Male	159	4.2	3636	95.8	0.997
Female	249	4.2	5692	95.8	
Marital status					
Single	15	4.5	320	95.5	0.675
Married	349	4.1	8163	95.9	
Widow	36	5	686	95	
Divorced	8	4.8	159	95.2	
Residence type					
Urban	272	4.5	5,728	95.5	0.032
Rural	136	3.6	3,600	96.4	
Education level					
Illiteracy	299	4.9	5815	95.1	0.001
Primary school	55	3.5	1538	96.5	
Secondary school	20	3.2	611	96.8	
High school diploma	22	3.1	680	96.9	
University	12	1.7	684	98.3	
Wealth status					
Poorest	96	4.9	1864	95.1	0.142
Poor	92	4.8	1880	95.2	
Moderate	79	4.1	1845	95.9	
Rich	75	3.8	1893	96.2	
Richest	66	3.5	1846	96.5	
Smoking status					
Smoker	117	5.9	1851	94.1	0.001
Nonsmoker	291	3.7	7477	96.3	
Pack year					
0	291	3.7	7477	96.3	0.001
0.1-5	38	6.9	516	93.1	
5.1-15	20	4.9	386	95.1	
15.1-30	24	5.4	422	94.6	
≥ 30	32	6.1	490	93.9	
History of stroke					
Yes	19	12.4	134	87.6	
No	389	4.1	9194	95.9	
History of epilepsy					
Yes	10	9.3	97	90.7	0.007
No	398	4.1	9231	95.9	
History of head trauma					
Yes	45	7.8	531	92.2	0.001
No	636	4	8797	96	

 The overall prevalence of amnesia was 4.2% (95% confidence interval [CI]: 3.8–4.6). The prevalence of amnesia increased with age (*P* for trend < 0.001). However, the overall prevalence rate of amnesia was not significantly different between females and males (*P*= 0.999). Additionally, no significant difference was detected between genders within any age group (*P*> 0.05). The highest prevalence rate of amnesia was 7.0% (95% CI: 4.8, 9.9) in women aged 65 years and older. The prevalence rates of amnesia stratified by gender and age are presented in Table 2.

**Table 2:** The prevalence rates and 95% CIs of amnesia in terms of age and gender

**Age group (y)**	**Male**			**Female**			**Total**			***P *** **value**
	**n**	**Cases**	**Prevalence (%)** **(95% CI)**	**n**	**Cases**	**Prevalence (%)** **(95% CI)**	**n**	**Cases**	**Prevalence (%)** **(95% CI)**	
35-39	653	14	2.0 (1.2–3.5)	1177	30	2.5 (1.8–3.6)	1830	44	2.4 (1.8–3.2)	0.588
40-44	739	29	3.9 (2.6–5.6)	1222	37	3.0 (2.1–4.1)	1961	66	3.4 (2.6–4.2)	0.286
45-49	673	32	4.8 (3.3–6.6)	1058	51	4.8 (3.6–6.3)	1758	83	4.8 (3.8–5.9)	0.958
50-54	586	24	4.1 (2.7–5.9)	867	43	5.0 (3.6–6.6)	1453	67	4.7 (3.8–5.8)	0.441
55-59	520	22	4.2 (2.7–6.3)	733	36	4.9 (3.5–6.7)	1253	58	4.7 (3.6–6.0)	0.572
60-64	349	24	6.9 (4.5–10.1)	432	22	5.1 (3.2–7.6)	781	46	5.9 (4.4–7.7)	0.292
≥ 65	275	14	5.1 (2.8–8.4)	425	30	7.1 (4.8 –9.9)	700	44	6.3 (4.6–8.3)	0.295
Total	3795	159	4.2 (3.6–4.9)	5941	249	4.2 (3.7–4.8)	9736	408	4.2 (3.7–4.7)	0.997

*Note*. CI: Confidence interval.

 The logistic regression model was performed with crude and adjusted ORs to evaluate the strength of associations and control for potential confounders. The ORs and their 95% CIs are listed in Table 3. The crude ORs showed statistical significance for all assessed variables, except for gender, marital status, and wealth status (*P*< 0.05). All variables with a *P* value < 0.25 were included in the multiple logistic regression models. In the final stage of the model, the adjusted ORs for age, education level, current smoking status, residence type, stroke, epilepsy, and history of head trauma were statistically significant (*P*< 0.05).

**Table 3:** Unadjusted and adjusted ORs and 95% CIs using the logistic regression model

**Variables**	**Unadjusted OR ** **(95% CI)**	***P*** ** value**	**Adjusted OR ** **(95% CI)**	***P*** ** value**
Age group (y)				
35-39	1.00		1.00	
40-44	1.32 (0.89–1.95)	0.050	1.32 (0.90–1.95)	0.160
45-49	1.72 (1.72–2.50)	0.001	1.72 (1.18–2.51)	0.005
50-54	1.58 (1.07–2.35)	0.001	1.59 (1.07–2.36)	0.021
55-59	1.45 (0.96–2.19)	0.001	1.46 (0.97–2.22)	0.070
60-64	1.83 (1.18–2.83)	0.001	1.84 (1.19–2.86)	0.006
≥ 65	1.88 (1.20–2.94)	0.001	1.90 (1.21–2.97)	0.005
Gender				
Female	1.00			
Male	1.01 (0.82–1.23)	0.972	-	-
Marital status				
Single	1.00			
Married	0.94 (0.55–1.59)	0.818	-	-
Widow	1.16 (0.63–2.14)	0.644	-	-
Divorced	1.09 (0.45–2.62)	0.853	-	-
Educational level				
Illiterate	2.86 (1.58–5.18)	0.001	2.85 (1.58–5.16)	0.001
Primary school	2.03 (1.08–3.83)	0.021	2.04 (1.08–3.85)	0.028
Middle school	1.82 (0.88–3.76)	0.041	1.83 (0.88–3.79)	0.104
High school	1.78 (0.87–3.64)	0.103	1.79 (0.88–3.65)	0.110
University	1.00		1.00	
Smoking				
No	1.00		1.00	
Yes	1.53 (1.21–1.92)	0.001	1.52 (1.21–1.91)	0.001
Pack year				
0	1.00		1.00	
0.1-5	1.90 (1.35–2.67)	0.001	1.85 (1.29–2.65)	0.001
5.1-15	1.26 (0.78–2.03)	0.338	1.24 (0.77–2.00)	0.378
15.1-30	1.37 (0.89–2.12)	0.156	1.35 (0.88–2.10)	0.174
> 30	1.77 (1.22 –2.59)	0.003	1.46 (0.99–2.16)	0.055
Residence type				
Rural	1.00		1.00	
Urban	1.44 (1.16–1.79)	0.001	1.41 (1.14–1.76)	0.002
Wealth status				
Poorest	1.00		1.00	
Poor	0.97 (0.72–1.31)	0.849	-	-
Moderate	0.83 (0.61–1.12)	0.216	-	-
Rich	0.77 (0.57–1.04)	0.090	-	-
Richest	0.69 (0.51–0.95)	0.023	-	-
History of stroke				
No	1.00		1.00	
Yes	2.61 (1.58–4.31)	0.001	2.67 (1.62–4.41)	0.001
History of epilepsy				
No	1.00		1.00	
Yes	2.05 (1.05–4.02)	0.036	2.07 (1.06–4.06)	0.034
History of head trauma				
No	1.00		1.00	
Yes	1.87 (1.35–2.60)	0.001	1.97 (1.42–2.73)	0.001

*Note*. CI: Confidence interval; OR: Odds ratio.

 The results (Table 3) indicated that the participants in the ≥ 65-year-old group had almost twofold higher odds of amnesia compared to those in the 35–39-year-old group [OR = 1.92 (95% CI: 1.23–2.98)]. Literacy had the strongest association with amnesia among the assessed factors, so that the odds of having amnesia were almost three times greater in illiterate participants than in those with university literacy [OR = 2.97 (95% CI: 1.64–5.37)]. Moreover, smokers had 57% higher odds of having amnesia than nonsmokers [OR = 1.57 (95% CI: 1.26–1.97)]. Urban residents had 42% higher odds of having amnesia than rural residents [OR = 1.42 (95% CI: 1.15–1.76)]. The odds of amnesia were nearly 2.5 times higher in people with a history of stroke compared to those without a history of stroke [OR = 2.43 (95% CI: 1.47–4.01)]. Additionally, having epilepsy increased the odds of amnesia twofold [OR = 2.02 (95% CI: 1.03–3.95)]. Finally, participants with a history of head trauma had 84% higher odds of amnesia than the other participants [OR = 1.84 (95% CI: 1.33–2.54)].

## Discussion

 This study explored the risk factors associated with amnesia and the relationship between smoking and amnesia. The main findings of our study confirmed a statistically significant relationship between smoking and amnesia. Additionally, several other factors, including age, education level, type of residence, history of stroke, epilepsy, and history of trauma, were associated with amnesia.

 The relationship between age and amnesia is complex and influenced by various cognitive, neural, and genetic factors. Age-related changes in the brain, such as reduced blood flow and neuron loss, significantly contribute to memory decline and amnesia. Research indicates that the hippocampus, crucial for memory formation, undergoes alterations with age, leading to cognitive deficits.^22^ Additionally, older adults are more susceptible to false memories due to cognitive decline, particularly in frontal functioning, which is crucial for memory accuracy.^23^

 In the present study, an inverse relationship was observed between education level and amnesia. More precisely, the odds of amnesia were lower in individuals with a higher education level. Several studies reported that education levels can impact cognitive function and memory, highlighting the potential protective effect of education and cognitive stimulation on cognitive decline and Alzheimer’s disease.^24,25^ People with higher education levels may have lower levels of amnesia due to cognitive reserve, lifestyle factors, social engagement, and cognitive stimulation.^26,27^

 Our results demonstrated that living in urban areas increased the odds of having amnesia. It is important to note that while these studies provide valuable insights, the relationship between type of residence and amnesia is complex and multifactorial, and more research is needed to fully understand this relationship.^28,29^ Community-based studies revealed that the prevalence of mild cognitive impairment was significantly higher in urban areas compared to rural areas. The researchers suggest that this difference may be due to variations in lifestyle and environmental factors between urban and rural areas.^30^

 In our study, while the odds of amnesia were slightly higher in poor people, the association between wealth index and amnesia was not statistically significant. Some studies indicated that individuals with lower SES are more likely to experience cognitive decline and memory problems than those with higher SES. This problem could be due to limited access to healthcare, lower education levels, or exposure to environmental toxins. Some studies have also reported that individuals with lower SES are more likely to experience stress, which can affect brain function and memory. Chronic stress can lead to changes in the hippocampus, a brain region involved in memory formation, and increase the risk of developing amnesia. However, it is noteworthy that the findings of each study may vary based on their sample size, methodology, and other factors.^31-33^ The findings of a study revealed that involvement in leisure activities, such as reading, playing games, and engaging in social activities, was linked to a reduced risk of dementia. However, in the mentioned study, no significant correlation was found between SES and the risk of dementia.^34^

 In the present study, the odds of amnesia were significantly higher in smokers than in nonsmokers. Smoking has been related to many adverse health effects, including cognitive impairment and an increased risk of developing dementia. While there is no direct link between smoking and amnesia, some studies have suggested that smoking may contribute to memory problems. Research has shown that smoking can cause damage to blood vessels in the brain and reduce blood flow to the brain, impairing cognitive function and memory. Additionally, smoking has been linked to oxidative stress and inflammation in the brain, which can also contribute to cognitive decline. Long-term heavy smoking has also been associated with an increased risk of developing Alzheimer’s disease, a type of dementia that causes memory loss and other cognitive problems. These studies concluded that smoking is related to cognitive impairments and memory problems in young and older adults. However, it is worth mentioning that smoking is one of many factors contributing to memory problems, and quitting smoking can help improve overall brain health.^35,36^ Several studies confirmed that smoking is associated with decreased cognitive function and memory problems. Ceasing smoking can help enhance overall brain health and reduce the risk of developing memory problems later in life.^37^ Additionally, another study revealed that smoking was related to reduced brain volume and white matter hyperintensities, which are linked to memory problems and cognitive impairments. In contrast to individuals who have never smoked, middle-aged males who smoke undergo a more rapid deterioration in cognitive ability, particularly in terms of global cognition and executive function. Conversely, individuals who have quit smoking for a minimum of ten years do not appear to experience any negative consequences of cognitive decline.^38^

 The relationship between smoking and TGA is complex, involving various mechanisms that can affect memory function, such as the impact of smoking on brain structure, particularly in the hippocampus, which is critical for memory formation.^39^ Cigarette smoke contains numerous harmful chemicals that contribute to oxidative stress in the brain.^40^ This stress can lead to cellular damage, inflammation, and neurodegeneration, further compromising memory function and increasing the risk of TGA episodes. Additionally, nicotine interacts with nicotinic acetylcholine receptors, affecting neurotransmitter systems involved in memory.^41^ Furthermore, nicotine stimulates the release of dopamine, which can create a pleasurable sensation but may also disrupt normal cognitive processes when chronically elevated.^41^ This disruption can lead to difficulties in attention and memory recall, contributing to episodes of amnesia.

 The present study evaluated the dose-response relationship between smoking and amnesia. Consistent with our findings, a meta-analysis examined prospective studies on the association between smoking and cognitive decline, encompassing dementia and amnesia. The researchers discovered that the risk of amnesia escalated with the daily number of cigarettes smoked. Moreover, the study indicated a decrease in the risk of cognitive decline among former smokers, implying that ceasing smoking could have a safeguarding effect on cognitive function.^42^ Furthermore, a review study discussed various mechanisms through which smoking might contribute to cognitive impairment, such as oxidative stress, inflammation, and vascular damage. This investigation offered proof of a dose-response connection between smoking and cognitive impairments, including amnesia, with heavier smokers displaying more pronounced cognitive deficits than lighter smokers. The researchers concluded that smoking constitutes a risk factor for cognitive impairments and that quitting smoking could potentially alleviate these risks.^43^ Nevertheless, some studies did not observe a link between smoking and forgetfulness.^44,45^

 In line with our results, the findings of a study suggested that head trauma can lead to a range of cognitive deficits, including amnesia, highlighting the importance of early diagnosis and rehabilitation for individuals with head injury-related cognitive impairments.^38^

 Our findings demonstrated a significant relationship between amnesia and stroke, which is in line with the results of another research.^7^ However, some studies reported no relationship between amnesia and stroke.^46-49^ This difference in conclusions can depend on the participant’s characteristics, study design, the follow-up period, and the sample size.

 Based on our results, there was a statistically significant association between a history of epilepsy and amnesia. Several studies suggested that individuals with chronic epilepsy may experience cognitive impairments, including deficits in memory and other cognitive domains, which conforms to the findings of the present study. In addition, the effects of epilepsy on memory may vary depending on the type and location of seizures, as well as the effects of antiepileptic drugs. These findings offer updated insights into the cognitive and neurological consequences of chronic epilepsy, including the impact of epilepsy on memory and the relationship between epilepsy and amnesia.^50,51^

 Our study had several strengths, including the large sample size, which can improve the reliability and generalizability of the results. The study was designed within the framework of a population-based cohort study, allowing for the generalization of results to the broader population. It was conducted on Arab ethnicity in southwest Iran. Given the similarities in ethnicity, customs, and lifestyle between Iranian Arabs and Arabs in neighboring countries, such as Iraq and Kuwait, the results can be extrapolated to a wide geographic area with millions of people. However, the current study had several limitations. This study had a cross-sectional design, which can only establish associations but not causality. In addition, genetic information was not collected in this study. Smoking status and history of pack-years were self-reported, which may have introduced bias as participants may have recalled or reported them differently in the groups with and without amnesia. Factors that can influence self-reported memory complaints in amnesia patients include personality traits, demographic factors, and affective symptoms (e.g., depressive symptoms). Additionally, cognitive impairments may result in changes in smoking behavior, rather than the other way around. For example, individuals with cognitive impairments may start smoking or be more inclined to smoke heavily.

**Highlights**

- The relationship between amnesia and smoking was statistically significant.
- Amnesia significantly increased with an increase in the pack years of cigarette consumption.
- Demographic risk factors of amnesia were age, education, and type of residence.
- Clinical risk factors of amnesia were stroke, epilepsy, and a history of trauma.
- No significant association was found between gender, marital, wealth index, and amnesia.

## Conclusion

 These findings confirmed a direct correlation between smoking and amnesia. Understanding the impact of smoking on amnesia can help health policymakers plan health improvement programs, including prevention and educational interventions. The association between smoking and amnesia is intricate and is expected to encompass numerous concurrent pathways. Therefore, future longitudinal studies will be necessary to better understand the mechanisms behind these associations.

## Acknowledgements

 We would like to thank the participants and staff of the Hoveyzeh Cohort Study Center who assisted us in conducting this study.

## Competing Interests

 The authors declare that they have no conflict of interests.

## Ethical Approval

 This project was approved by the Ethics Committee of Ahvaz Jundishapur University of Medical Sciences (IR AJUMS.REC.1398.361). The researchers adhered to the Helsinki Declaration and its later amendments during the study. Before the interviews and access to administrative records, all participants provided their consent by signing informed written consent forms.

## Funding

 This work was financially supported by the Research Deputy of Ahvaz Jundishapur University of Medical Sciences, Ahvaz, Iran (Grant number: HCS-9811). The Vice-Chancellor for Research at Ahvaz Jundishapur University of Medical Sciences, as our funding body, played no role in the study design, data collection, analysis, and interpretation, or manuscript writing.

## References

[1] Miller TD, Butler CR (2022). Acute-onset amnesia: transient global amnesia and other causes. Pract Neurol.

[2] Perrotta G (2020). Amnesia: definition, main models, classifications, neurobiological profiles and clinical treatments. Arch Depress Anxiety.

[3] Michaelson NM, Friedman SA, Ch’ang JH (2023). Update on transient global amnesia (TGA): current theories underlying the etiology, diagnosis, prognosis, and management of TGA. Curr Treat Options Neurol.

[4] Darshini JK, Afsar M, Vandana VP, Shukla D, Rajeswaran J (2021). The triad of cognition, language, and communication in traumatic brain injury: a correlational study. J Neurosci Rural Pract.

[5] Rao R, Topiwala A (2020). Alcohol use disorders and the brain. Addiction.

[6] Komulainen T, Bärlund V, Tanila H, Koivisto A, Jäkälä P (2023). Incidence and risk factors of transient global amnesia. Neuroepidemiology.

[7] Lee SH, Kim KY, Lee JW, Park SJ, Jung JM (2022). Risk of ischaemic stroke in patients with transient global amnesia: a propensity-matched cohort study. Stroke Vasc Neurol.

[8] Wu P, Li W, Cai X, Yan H, Chen M (2020). Associations of cigarette smoking with memory decline and neurodegeneration among cognitively normal older individuals. Neurosci Lett.

[9] Lewis CR, Talboom JS, De Both MD, Schmidt AM, Naymik MA, Håberg AK (2021). Smoking is associated with impaired verbal learning and memory performance in women more than men. Sci Rep.

[10] Middlekauff HR, Park J, Moheimani RS (2014). Adverse effects of cigarette and noncigarette smoke exposure on the autonomic nervous system: mechanisms and implications for cardiovascular risk. J Am Coll Cardiol.

[11] Sohel N, Tuokko H, Griffith L, Raina P (2016). Factors influencing discrepancies in self-reported memory and performance on memory recall in the Canadian Community Health Survey-Healthy Aging, 2008-09. Age Ageing.

[12] Hajdusianek W, Żórawik A, Waliszewska-Prosół M, Poręba R, Gać P (2021). Tobacco and nervous system development and function-new findings 2015-2020. Brain Sci.

[13] Mazzone P, Tierney W, Hossain M, Puvenna V, Janigro D, Cucullo L (2010). Pathophysiological impact of cigarette smoke exposure on the cerebrovascular system with a focus on the blood-brain barrier: expanding the awareness of smoking toxicity in an underappreciated area. Int J Environ Res Public Health.

[14] Guirguis S. The Acute Effects of Nicotine and Exercise on Working Memory [dissertation]. The University of Western Ontario; 2016. Available from: https://ir.lib.uwo.ca/etd/4081.

[15] Cansino S, Torres-Trejo F, Estrada-Manilla C, Hernández-Ramos E, Martínez-Galindo JG, Gómez-Fernández T (2018). Mediators of episodic memory decay across the adult life span. Sci Rep.

[16] Mohammadian M, Sarrafzadegan N, Roohafza HR, Sadeghi M, Hasanzadeh A, Rejali M (2018). A comparative study on the prevalence and related factors of cigarette smoking in Iran and other Asian countries: results of Isfahan Cohort Study (ICS). World Cancer Res J.

[17] Cheraghian B, Hashemi SJ, Hosseini SA, Poustchi H, Rahimi Z, Sarvandian S (2020). Cohort profile: the Hoveyzeh Cohort Study (HCS): a prospective population-based study on non-communicable diseases in an Arab community of Southwest Iran. Med J Islam Repub Iran.

[18] Poustchi H, Eghtesad S, Kamangar F, Etemadi A, Keshtkar AA, Hekmatdoost A (2018). Prospective epidemiological research studies in Iran (the PERSIAN Cohort Study): rationale, objectives, and design. Am J Epidemiol.

[19] Smits J, Steendijk R (2015). The international wealth index (IWI). Soc Indic Res.

[20] Hjelm L, Mathiassen A, Miller D, Wadhwa A. Creation of a Wealth Index. United Nations World Food Programme; 2017.

[21] Hecht SS (1999). Tobacco smoke carcinogens and lung cancer. J Natl Cancer Inst.

[22] Dahan L, Rampon C, Florian C (2020). Age-related memory decline, dysfunction of the hippocampus and therapeutic opportunities. Prog Neuropsychopharmacol Biol Psychiatry.

[23] West JT, Wagner RL, Steinkrauss A, Dennis N. Investigating the cognitive correlates of semantic and perceptual false memory in older and younger adults: a multi-group latent variable approach [Preprint]. 2024. 10.31234/osf.io/usr7n. PMC11951306 40160539

[24] Ngandu T, Lehtisalo J, Solomon A, Levälahti E, Ahtiluoto S, Antikainen R (2015). A 2-year multidomain intervention of diet, exercise, cognitive training, and vascular risk monitoring versus control to prevent cognitive decline in at-risk elderly people (FINGER): a randomised controlled trial. Lancet.

[25] Andel R, Vigen C, Mack WJ, Clark LJ, Gatz M (2006). The effect of education and occupational complexity on rate of cognitive decline in Alzheimer’s patients. J Int Neuropsychol Soc.

[26] Valenzuela MJ, Sachdev P (2006). Brain reserve and dementia: a systematic review. Psychol Med.

[27] Stern Y (2009). Cognitive reserve. Neuropsychologia.

[28] Huang WT, Li CY, Lee JT (2015). The association between residential types and dementia in Taiwan: a 13-year population-based cohort study. Int J Geriatr Psychiatry.

[29] Li CH, Chen WC, Liao WC, Tu CY, Lin CL, Sung FC (2015). The association between chronic obstructive pulmonary disease and Parkinson’s disease: a nationwide population-based retrospective cohort study. QJM.

[30] Jiang B, Liu Q, Li JP, Lin SN, Wan HJ, Yu ZW (2024). Prevalence and risk factors for dementia and mild cognitive impairment among older people in Southeast China: a community-based study. BMC Geriatr.

[31] Wang K, Fang Y, Zheng R, Zhao X, Wang S, Lu J (2024). Associations of socioeconomic status and healthy lifestyle with incident dementia and cognitive decline: two prospective cohort studies. EClinicalMedicine.

[32] Zahodne LB, Glymour MM, Sparks C, Bontempo D, Dixon RA, MacDonald SW (2011). Education does not slow cognitive decline with aging: 12-year evidence from the Victoria Longitudinal Study. J Int Neuropsychol Soc.

[33] Rawtaer I, Mahendran R, Kua EH, Tan HP, Tan HX, Lee TS (2020). Early detection of mild cognitive impairment with in-home sensors to monitor behavior patterns in community-dwelling senior citizens in Singapore: cross-sectional feasibility study. J Med Internet Res.

[34] Akbaraly TN, Portet F, Fustinoni S, Dartigues JF, Artero S, Rouaud O (2009). Leisure activities and the risk of dementia in the elderly: results from the Three-City Study. Neurology.

[35] Chamberlain SR, Odlaug BL, Schreiber LR, Grant JE (2012). Association between tobacco smoking and cognitive functioning in young adults. Am J Addict.

[36] Starr JM, Deary IJ, Fox HC, Whalley LJ (2007). Smoking and cognitive change from age 11 to 66 years: a confirmatory investigation. Addict Behav.

[37] Zaninotto P, Batty GD, Allerhand M, Deary IJ (2018). Cognitive function trajectories and their determinants in older people: 8 years of follow-up in the English Longitudinal Study of Ageing. J Epidemiol Community Health.

[38] Sabia S, Elbaz A, Dugravot A, Head J, Shipley M, Hagger-Johnson G (2012). Impact of smoking on cognitive decline in early old age: the Whitehall II cohort study. Arch Gen Psychiatry.

[39] Dobric A, De Luca SN, Seow HJ, Wang H, Brassington K, Chan SM (2022). Cigarette smoke exposure induces neurocognitive impairments and neuropathological changes in the hippocampus. Front Mol Neurosci.

[40] Kuntic M, Oelze M, Steven S, Kröller-Schön S, Stamm P, Kalinovic S (2020). Short-term e-cigarette vapour exposure causes vascular oxidative stress and dysfunction: evidence for a close connection to brain damage and a key role of the phagocytic NADPH oxidase (NOX-2). Eur Heart J.

[41] Soni S, Verma L (2024). Nicotine and neurotransmitters an update. Res J Pharm Technol.

[42] Anstey KJ, von Sanden C, Salim A, O’Kearney R (2007). Smoking as a risk factor for dementia and cognitive decline: a meta-analysis of prospective studies. Am J Epidemiol.

[43] Durazzo TC, Meyerhoff DJ, Nixon SJ (2010). Chronic cigarette smoking: implications for neurocognition and brain neurobiology. Int J Environ Res Public Health.

[44] Arena JE, Brown RD, Mandrekar J, Rabinstein AA (2017). Long-term outcome in patients with transient global amnesia: a population-based study. Mayo Clin Proc.

[45] Alessandro L, Calandri IL, Suarez MF, Heredia ML, Chaves H, Allegri RF (2019). Transient global amnesia: clinical features and prognostic factors suggesting recurrence. Arq Neuropsiquiatr.

[46] Jovanovic ZB, Pavlovic AM, Vujisic Tesic BP, Pekmezovic TP, Kostic Boricic MV, Cvitan EZ (2018). Comprehensive ultrasound assessment of the craniocervical circulation in transient global amnesia. J Ultrasound Med.

[47] Mangla A, Navi BB, Layton K, Kamel H (2014). Transient global amnesia and the risk of ischemic stroke. Stroke.

[48] Romoli M, Tuna MA, McGurgan I, Li L, Giannandrea D, Eusebi P (2019). Long-term risk of stroke after transient global amnesia in two prospective cohorts. Stroke.

[49] Garg A, Limaye K, Shaban A, Adams HP Jr, Leira EC (2021). Transient global amnesia does not increase the risk of subsequent ischemic stroke: a propensity score-matched analysis. J Neurol.

[50] Sen A, Capelli V, Husain M (2018). Cognition and dementia in older patients with epilepsy. Brain.

[51] Martin RC, Griffith HR, Faught E, Gilliam F, Mackey M, Vogtle L (2005). Cognitive functioning in community dwelling older adults with chronic partial epilepsy. Epilepsia.

